# Macrophages inhibit human osteosarcoma cell growth after activation with the bacterial cell wall derivative liposomal muramyl tripeptide in combination with interferon-γ

**DOI:** 10.1186/1756-9966-33-27

**Published:** 2014-03-10

**Authors:** Jens HW Pahl, Kitty MC Kwappenberg, Eleni M Varypataki, Susy J Santos, Marieke L Kuijjer, Susan Mohamed, Juul T Wijnen, Maarten JD van Tol, Anne-Marie Cleton-Jansen, R Maarten Egeler, Wim Jiskoot, Arjan C Lankester, Marco W Schilham

**Affiliations:** 1 Department of Pediatrics, Leiden University Medical Center, Leiden, the Netherlands; 2 Division of Drug Delivery Technology, Leiden Academic Center for Drug Research, Leiden University, Leiden, the Netherlands; 3 Department of Pathology, Leiden University Medical Center, Leiden, the Netherlands; 4 Department of Clinical Genetics, Leiden University Medical Center, Leiden, the Netherlands; 5 Division of Hematology/Oncology, Hospital for Sick Children/University of Toronto, Toronto, Canada; 6 Laboratory for Immunology, P3-P, Department of Pediatrics, Leiden University Medical Center, PO Box 9600, 2300 RC Leiden, the Netherlands

**Keywords:** Macrophages, Muramyl tripeptide, IFN-γ, Osteosarcoma, Cetuximab

## Abstract

**Background:**

In osteosarcoma, the presence of tumor-infiltrating macrophages positively correlates with patient survival in contrast to the negative effect of tumor-associated macrophages in patients with other tumors. Liposome-encapsulated muramyl tripeptide (L-MTP-PE) has been introduced in the treatment of osteosarcoma patients, which may enhance the potential anti-tumor activity of macrophages. Direct anti-tumor activity of human macrophages against human osteosarcoma cells has not been described so far. Hence, we assessed osteosarcoma cell growth after co-culture with human macrophages.

**Methods:**

Monocyte-derived M1-like and M2-like macrophages were polarized with LPS + IFN-γ, L-MTP-PE +/− IFN-γ or IL-10 and incubated with osteosarcoma cells. Two days later, viable tumor cell numbers were analyzed. Antibody-dependent effects were investigated using the therapeutic anti-EGFR antibody cetuximab.

**Results:**

M1-like macrophages inhibited osteosarcoma cell growth when activated with LPS + IFN-γ. Likewise, stimulation of M1-like macrophages with liposomal muramyl tripeptide (L-MTP-PE) inhibited tumor growth, but only when combined with IFN-γ. Addition of the tumor-reactive anti-EGFR antibody cetuximab did not further improve the anti-tumor activity of activated M1-like macrophages. The inhibition was mediated by supernatants of activated M1-like macrophages, containing TNF-α and IL-1β. However, specific blockage of these cytokines, nitric oxide or reactive oxygen species did not inhibit the anti-tumor effect, suggesting the involvement of other soluble factors released upon macrophage activation. While LPS + IFN-γ–activated M2-like macrophages had low anti-tumor activity, IL-10–polarized M2-like macrophages were able to reduce osteosarcoma cell growth in the presence of the anti-EGFR cetuximab involving antibody-dependent tumor cell phagocytosis.

**Conclusion:**

This study demonstrates that human macrophages can be induced to exert direct anti-tumor activity against osteosarcoma cells. Our observation that the induction of macrophage anti-tumor activity by L-MTP-PE required IFN-γ may be of relevance for the optimization of L-MTP-PE therapy in osteosarcoma patients.

## Introduction

Osteosarcoma is the most frequent malignant bone tumor in adolescents and young adults. Of patients with localized, non-metastatic disease, up to 70% achieve persistent remission [1]. In contrast, prognosis of patients with advanced, metastatic and recurrent disease is as low as 20% despite intensive chemotherapy and surgery. Thus, novel therapies are needed, especially for patients with chemotherapy-resistant disease [2,3]. Recently, we have demonstrated that the presence of tumor-infiltrating macrophages at the time of diagnosis is positively correlated with a favorable outcome of patients with osteosarcoma [4]. Hence, targeting tumor-associated macrophages in osteosarcoma with macrophage-activating agents is an attractive option to complement current anti-tumor treatments.

Macrophages are mononuclear phagocytic cells that are involved in homeostatic, pro-inflammatory and immune regulatory responses in the tissue [5,6]. While macrophages can originate from blood monocytes under inflammatory conditions, as in the classical model for macrophage development, it has recently been revealed that under non-inflammatory conditions tissue macrophages primarily originate from the yolk sac and fetal liver and are maintained independently of hematopoietic precursors [7]. Macrophages possess great functional and phenotypic plasticity which is often simplified by classification in M1 and M2 phenotypes [8]. M1 macrophages are involved in host defense through their bactericidal and tumoricidal activity and pro-inflammatory cytokine production if ‘classically-activated’ by interferon-γ (IFN-γ) and Toll-like receptor ligands such as bacterial lipopolysaccharide (LPS) [9,10]. M2 macrophages can exhibit many different phenotypes in response to diverse stimuli such as polarization with interleukin-10 (IL-10) or LPS. M2 macrophages are involved in scavenging cell debris and bacteria, antibody-dependent phagocytosis, tissue remodeling, angiogenesis, wound healing and immune regulation. In contrast to ‘classically-activated’ M1 macrophages, macrophages with an M2-like phenotype are often detected in solid tumors and considered to promote tumor progression [8-11].

Macrophages constitute the majority of tumor-infiltrating immune cells in solid tumors including osteosarcoma [4,12]. In most tumors, the presence of macrophages represents an unfavorable prognostic factor [13]. In contrast, in osteosarcoma as well as colorectal cancer higher numbers of tumor-infiltrating macrophages correlate with better survival [4,14,15]. In osteosarcoma, there was no clear association of good survival with an M1-like or M2-like phenotypic polarization of macrophages [4].

Monocytes and macrophages activated with LPS have been implicated in anti-tumor responses for a long time [16-21]. But while canine macrophages have been reported to have anti-tumor activity against canine osteosarcoma cells, comparable evidence for anti-tumor activity of human macrophages against human osteosarcoma cells is not available. The anti-tumor activity of canine macrophages was shown to be dependent on stimulation with LPS or another bacterial cell wall constituent, i.e. muramyl dipeptide (MDP) or the lipophilic derivative muramyl tripeptide phoshatidylethanolamine (MTP-PE) [22]. Application of liposome-encapsulated MTP-PE (L-MTP-PE) *in vivo* improved survival of dogs with osteosarcoma [23]. This observation encouraged the addition of L-MTP-PE to the treatment of osteosarcoma patients as a macrophage-activating agent but did not increase event-free survival of non-metastatic or metastatic osteosarcoma patients [1,24].

Therefore, we set out to investigate the anti-tumor activity of human macrophages against human osteosarcoma cells and determine whether this activity can be manipulated. We set up an *in vitro* model in which the effect of human macrophages on the growth of osteosarcoma cells can be directly assessed by counting residual tumor cells after a two-day co-culture with macrophages. Using this model we demonstrate how anti-tumor activity of M1-like macrophages and M2-like macrophages can be induced by bacterial stimuli like L-MTP-PE and the therapeutic anti-EGFR antibody cetuximab, respectively.

## Materials and methods

### Cell lines

The osteosarcoma cell lines HOS, HOS-143b, OHS, OSA, SAOS-2 and U2OS were obtained from the EuroBoNeT cell line repository (2007) [25]. Cell line identity was confirmed by short tandem repeat DNA fingerprinting in 2012. All cell lines were maintained in RPMI 1640 (Invitrogen, Carlsbad, CA, USA) supplemented with 10% fetal calf serum (Invitrogen) and 100 U/ml penicillin and 100 ug/ml streptomycin (Invitrogen). All cell lines were negative for mycoplasma infection as regularly tested by RT-PCR.

### Preparation of liposomal MTP-PE

Liposomes (multi-lamellar vesicles) were prepared from a mixture of the synthetic phospholipids 1-palmitoyl-2-oleoyl-sn-glycero-3-phosphocholine (POPC, 850457P) and 1,2-dioleoyl-sn-glycero-3-phospho-L-serine (DOPS, 840035P) (both from Avanti Polar Lipids, Alabaster, Al, USA) at a 7:3 molar ratio in chloroform by mechanical agitation on a vortex mixer. MTP-PE (M_r_ 1237.5 g/mol; Mifamurtide; Sigma-Aldrich, St. Louis, MO, USA) was dissolved in chloroform:methanol:water 60:36:4 (v/v/v). 5 mg of liposomes (M_r_ 775 g/mol) were loaded with 0.02 mg of MTP-PE (1:250 ratio). The organic solution was dried in a rotary evaporator under reduced pressure for one hour to obtain a dry lipid film. Afterwards, the lipid film was rehydrated in 2.5 ml sterile PBS, resulting in a final concentration of 6.45 nmol MTP-PE per 2 μmol/ml liposome preparation (L-MTP-PE). The liposomes were passed four times through a 1 μm unipore polycarbonate filter (Nuleopore). Empty control liposomes (L-PBS) were prepared by the same procedure except without MTP-PE addition. The z-average diameter of the liposomes was ~350 nm with a mean zeta potential of −97 mV as measured on a Zetasizer (version 6.01) (Malvern Instruments, Worcestershire, UK).

### Monocyte Isolation and differentiation to macrophages

PBMC were separated from buffy coats of healthy adult donors (Sanquin Blood bank, Region Southwest, Rotterdam, the Netherlands) by Ficoll-Hypaque density gradient centrifugation. Monocytes were isolated from PBMC by positive selection using anti-CD14 MicroBeads (Miltenyi Biotech, Bergisch Gladbach, Germany). For M1-like and M2-like macrophage differentiation, monocytes (1,5 × 10^6^ per well per 3 ml of a 6-well tissue culture plate) were incubated with GM-CSF (80 ng/ml; Peprotech, Rocky Hill, NJ, USA) and M-CSF (20 ng/ml, R&D Systems, Minneapolis, MN, USA) for seven days as previously established [10,26]. In some conditions, M1-like and M2-like macrophages were additionally stimulated during the last day of differentiation with combinations of LPS (10 ng/ml; *E. coli* strain 0111:B4; Sigma-Aldrich), IFN-γ (100 U/ml; Boehringer, Mannheim, Germany), empty control liposomes (250 nmol) (L-PBS) or liposomes (250 nmol) containing MTP-PE (0.8 nmol, i.e., 1 μg) (L-MTP-PE) per 3 ml culture medium. M2-like macrophages were alternatively stimulated with IL-10 (10 ng/ml; Peprotech) during the last two days of differentiation. The phenotype of macrophage populations was tested in each experiment. Macrophages were devoid of the monocyte-derived dendritic cell marker CD1a (data not shown).

### Macrophage-tumor cell co-cultures

After seven-day differentiation, culture supernatants of macrophages were collected. Adherent macrophages were washed in cold PBS, detached by incubation in accutase (Sigma-Aldrich) for 30 min at 37°C and combined with the non-adherent cell fraction. Cell scraping of firmly adherent macrophages was avoided to maximize macrophage viability. Macrophages were seeded in 96-well flat-bottom plates in RPMI medium at 3,000 (cell conjugate formation assay) or 30,000 cells (tumor cell survival assay) per well (four wells per condition) and incubated for cell attachment. After two hours 3,000 osteosarcoma cells were added and macrophage-tumor cell co-cultures were incubated for two hours in cell conjugate formation assays at a 1:1 ratio in 50 μl medium and for two hours, one day and two days in tumor cell survival assays at a 10:1 ratio in 100 μl medium. In some experiments, tumor cells were coated with the chimeric monoclonal antibody cetuximab (anti-epidermal growth factor receptor, 1 μg/ml final concentration in co-cultures; Erbitux; Merck KGaA, Darmstadt, Germany) or with the non-binding anti-CD20 antibody rituximab (1 μg/ml; MabThera; Roche, Basel, Switzerland) prior to the co-culture. In blocking experiments, co-cultures were performed in the presence of the soluble tumor-necrosis factor-α (TNF-α) receptor etanercept (10 μg/ml; Enbrel; Wyeth; Madison, NJ, USA) and TNF-α neutralizing antibody adalimumab (10 μg/ml; Humira; Abbot; North Chicago, IL, USA), the IL-1 receptor antagonist anakinra (10 μg/ml; Kineret; Amgen; Thousand Oaks, CA, USA), nitric oxide species inhibitor Nω-Nitro-L-arginine methyl ester (10 μM; L-NAME; Sigma-Aldrich), reactive oxygen species inhibitors catalase (186 μg/ml; Sigma-Aldrich) and superoxide dismutase (4.2 μg/ml; Sigma-Aldrich).

### Anti-tumor activity assay

The effect of macrophages on tumor cell survival was assessed by enumerating tumor cells by flow cytometry [15,27]. Adherent and non-adherent cells were harvested after co-culture using accutase (if necessary supported by cell scraping) and stained with anti-CD56 and anti-CD32 to distinguish tumor cells and macrophages, respectively. The complete tumor cell-macrophage suspension was analyzed by flow cytometry. Live-gated tumor cells present at the end of the co-culture were quantified and in each experiment compared to the number of tumor cells grown in the absence of macrophages. In some experiments viable tumor cell numbers were measured after their incubation in medium with 50% (v/v) of macrophage cell-free supernatant or after their incubation with inhibitors in the presence of macrophages. Single measurements from multiple independent experiments were combined as indicated in figure legends.

### Cell conjugate formation

Tumor cell lines were labeled with CFSE (1 μM; Invitrogen) and incubated overnight to allow leakage of excess CFSE. IL-10–stimulated M2-like Macrophages were co-cultured with CFSE-labeled HOS-143b cells for two hours at 1:1 ratio. All cells were harvested from the culture by cell scraping and macrophages were labeled with APC-labeled anti-CD32 antibodies. Cell conjugate formation between macrophages and tumor cells was analyzed by flow cytometry, assessing the percentage of CD32^+^ macrophages acquiring high CFSE fluorescence from tumor cells.

For an indication of phagocytosis, after the cell conjugate formation assay, CD32^+^ macrophages which have acquired the fluorescent signal of CFSE^+^ tumor cells were sorted by flow cytometry in one experiment. The cells were stained with mouse anti-human HLA-DR (TAL.1B5; Dako, Glostrup, Denmark) followed by the Alexa-Fluor-594 goat anti-mouse IgG1 secondary antibody (Invitrogen) and embedded in Vectashield mounting medium containing DAPI (Vectorlabs, Burlingame, CA, USA). Cell conjugates were examined with a Leica DM5000 fluorescence microscope and LAS-AF acquisition program (Leica, Solms, Germany), detecting nuclei in blue, HLA-DR^+^ macrophages in red and CFSE^+^ tumor cells in green.

### Flow cytometry

The following fluorochrome-labeled mouse anti-human monoclonal antibodies were used: CD32^APC^ (clone FLI8.26), CD86^PE^ (FUN-1), CD163^PE^ (GHI/61), HLA-DR^FITC^ (L243) (BD Biosciences, Franklin Lakes, NJ, USA); CD56^PE^ (NKH-1) (Beckman Coulter, Brea, CA, USA); CD16^FITC^ (3G8), CD64^FITC^ (22) (IOTEST Immunotech, Marseille, France). Measurements were performed with the FACSCalibur (BD Biosciences) and analyzed with the BD Cell Quest ProTM software (version 5.2.1). Fold-expression data indicated in histogram plots were calculated by dividing the geometric mean fluorescence intensity (geoMFI) of antibodies by the geoMFI of the PBS control.

### Luminex assay

Cytokine production in cell-free supernatants of macrophage cultures was measured using the Bio-Plex Pro Human Cytokine 27-plex group 1 panel according to the manufacturer’s description (Bio-Rad Laboratories, Hercules, CA, USA).

### Statistical analysis

Paired student t-tests were performed to compare the means two samples. One-way analysis of variance (ANOVA) was performed to compare the means of three or more samples followed by Dunnett’s or Bonferroni’s multiple comparison post test to compare samples of interest with a control sample as described in the figure legends. Error bars represent the standard error of the mean. A P-value of <0.05 was considered statistically significant. All statistical analyses were performed using Graphpad Prism version 5.04 (La Jolla, CA, USA).

## Results

### M1-like macrophages inhibit osteosarcoma cell growth if activated with LPS + IFN-γ

**Figure 1:**
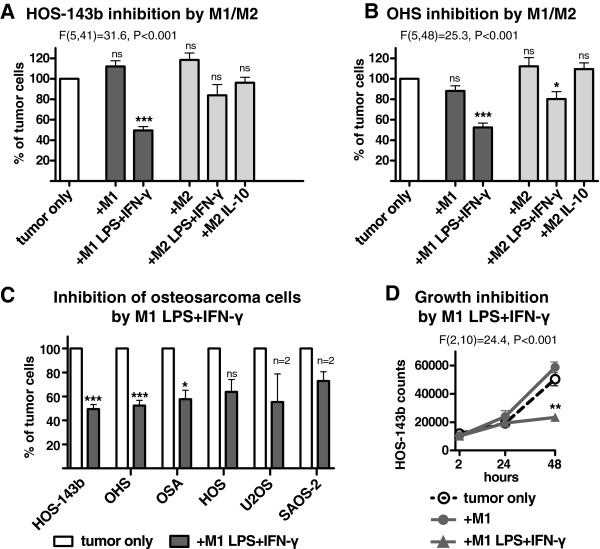
**Inhibition of osteosarcoma cell growth by LPS + IFN-γ–activated M1-like macrophages.** Human M1-like macrophages (dark shade) and M2-like macrophages (light shade) were pre-activated with or without LPS + IFN-γ (M1 and M2) or IL-10 (M2) and afterwards incubated with **(A)** HOS-143b cells (n = 4–11) and **(B)** OHS cells (n = 5–12) and **(C)** four other osteosarcoma cell lines (n = 2–12). After two days of co-culture, tumor cell numbers were counted by flow cytometry. Differences between one macrophage–tumor co-culture and the control, i.e. tumor cell recovery after incubation in the absence of macrophages (white bar, set to 100%), as in panel C were statistically analyzed by paired student t-tests, *** is P < 0.001, ** is P < 0.01, * is P < 0.05, ns is not statistically significant. Differences between multiple groups as in panel A and B were statistically analyzed by ANOVA as indicated followed by Dunnett’s post test for differences (p < 0.05) between individual co-cultures and the control as indicated by asterisks. **(D)** HOS-143b cell counts after 2, 24 and 48 hours co-culture with M1-like macrophages +/− LPS + IFN-γ (n = 5). All data are means of multiple experiments as indicated (n).

The potential of human macrophages to inhibit osteosarcoma cell growth *in vitro* was investigated. M1-like and M2-like macrophages were differentiated from blood monocytes with or without the polarization stimuli LPS + IFN-γ (for M1 and M2) or IL-10 (for M2) as previously established [10,26,28]. The various macrophage subtypes were co-cultured with osteosarcoma cell lines and after two days the residual number of viable tumor cells was assessed by flow cytometry [15,27]. In particular M1-like macrophages pre-stimulated with LPS + IFN-γ were able to significantly reduce tumor cell numbers of HOS-143b and OHS cells to as low as 50% and to lesser extend of four other osteosarcoma cell lines (Figure 1, panel A-C). The inhibition of tumor cell growth as a consequence of macrophage addition was not yet apparent after 2 and 24 hours of co-culture but became pronounced after two days of co-culture (Figure 1, panel D). The inhibitory effect of activated M1-like macrophages could be titrated and was near-maximal at >6:1 ratio (Additional file 1: Figure S1, panel A).

M2-like macrophages stimulated with LPS + IFN-γ showed less anti-tumor activity than LPS + IFN-γ–stimulated M1-like macrophages, while IL-10–polarized M2-like macrophages were not able to inhibit tumor cell growth (Figure 1, panel A and B). Incubation of tumor cells with LPS + IFN-γ alone had no inhibiting effect (data not shown).

**Figure 2:**
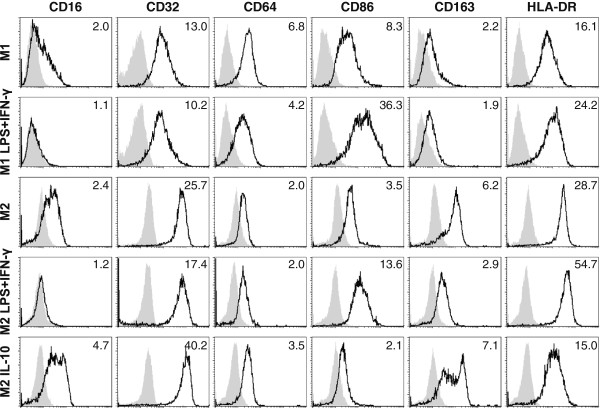
**Phenotypes of M1-like and M2-like macrophages.** Surface expression of Fcγ receptors (CD16, CD32 and CD64), HLA-DR, CD86 and CD163 on the various M1-like and M2-like macrophage populations was analyzed by flow cytometry. Fold change of geoMFI of specific antibody stainings (bold solid line) compared to PBS control (light shade) is indicated in the FACS histogram plots. Representative data of three experiments are depicted.

Induction of anti-tumor activity by M1-like macrophages after stimulation with LPS + IFN-γ was associated with a more activated phenotype as indicated by the up-regulation of CD86 and HLA-DR expression (Figure 2). In contrast to M1-like macrophages, M2-like macrophages expressed CD163, a marker frequently described for tumor-associated M2-like macrophages. LPS + IFN-γ–stimulated M2-like macrophages showed phenotypic similarities to LPS + IFN-γ–stimulated M1-like macrophages, exhibiting reduced levels of CD163 and increased levels of CD86 and HLA-DR expression. Notably, in particular when stimulated with IL-10, M2-like macrophages displayed high levels of FcγRII (CD32) in addition to FcγRI (CD64) and FcγRIIIa (CD16), suggesting that IL-10–polarized M2-like macrophages could exert antibody-dependent functions as described below.

Overall, of the different macrophage populations tested, LPS + IFN-γ–activated M1-like macrophages, resembling ‘classically-activated’ M1-like macrophages [8-10], were the most capable of inhibiting osteosarcoma cell growth.

### Liposomal muramyl tripeptide only induces anti-tumor activity of M1-like macrophages in the presence of IFN-γ

**Figure 3:**
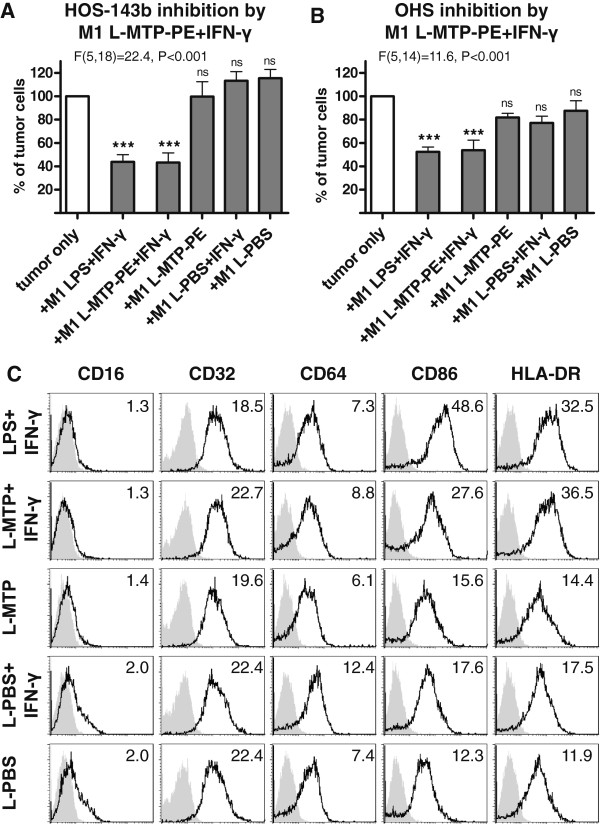
**M1-like macrophages stimulated with L-MTP-PE require IFN-γ for anti-tumor activity.** M1-like Macrophages were activated with LPS + IFN-γ, L-MTP-PE + IFN-γ, L-MTP-PE alone, L-PBS + IFN-γ or L-PBS alone. Tumor cell numbers of **(A)** HOS-143b (n = 3–5) and **(B)** OHS (n = 2–4) were assessed after two-day co-culture with activated macrophages and statistically analyzed relative to the control by ANOVA and Dunnett’s post test as indicated. **(C)** Surface expression of Fcγ receptors (CD64, CD32 and CD16), HLA-DR and CD86 was analyzed on M1-like macrophages after activation with LPS + IFN-γ, L-MTP-PE + IFN-γ, L-MTP-PE alone, L-PBS + IFN-γ or L-PBS alone. Representative data of two experiments are depicted.

In clinical applications, infusion of macrophage-activating bacterial products can cause severe, detrimental toxic reactions. This can be circumvented by incorporation of very lipophilic, synthetic MTP-PE into liposomes (L-MTP-PE), which results in rapid uptake by myeloid cells and low toxicity [21]. In spite of the inclusion of L-MTP-PE in clinical trials of osteosarcoma patients, direct evidence of L-MTP-PE to induce anti-tumor activity by human macrophages against human osteosarcoma cells is lacking. Therefore, it was investigated whether M1-like macrophages can be activated by L-MTP-PE to reduce tumor cell growth. Remarkably, only when co-stimulated with IFN-γ, L-MTP-PE–stimulated M1-like macrophages significantly inhibited tumor cell growth such as of HOS-143b cells and OHS cells to as low as 45% (Figure 3, panel A and B). The inhibition by L-MTP-PE + IFN-γ–stimulated M1-like macrophages was as potent as by LPS + IFN-γ–activated M1-like macrophages. In contrast, M1-like macrophages stimulated with MTP-PE-loaded liposomes alone, empty liposomes (L-PBS) or empty liposomes in combination with IFN-γ failed to inhibit tumor cell growth. Moreover, only when co-stimulated with IFN-γ, L-MTP-PE–stimulated M1-like macrophages exhibited an activated phenotype by CD86 and HLA-DR up-regulation similar to LPS + IFN-γ–activated M1-like macrophages (Figure 3, panel C). In conclusion, L-MTP-PE stimulation induced substantial anti-tumor activity of M1-like macrophages but only after co-stimulation with the pro-inflammatory cytokine IFN-γ.

### Soluble factors produced by M1-like macrophages after LPS + IFN-γ and L-MTP-PE + IFN-γ activation inhibit tumor cell growth

**Figure 4:**
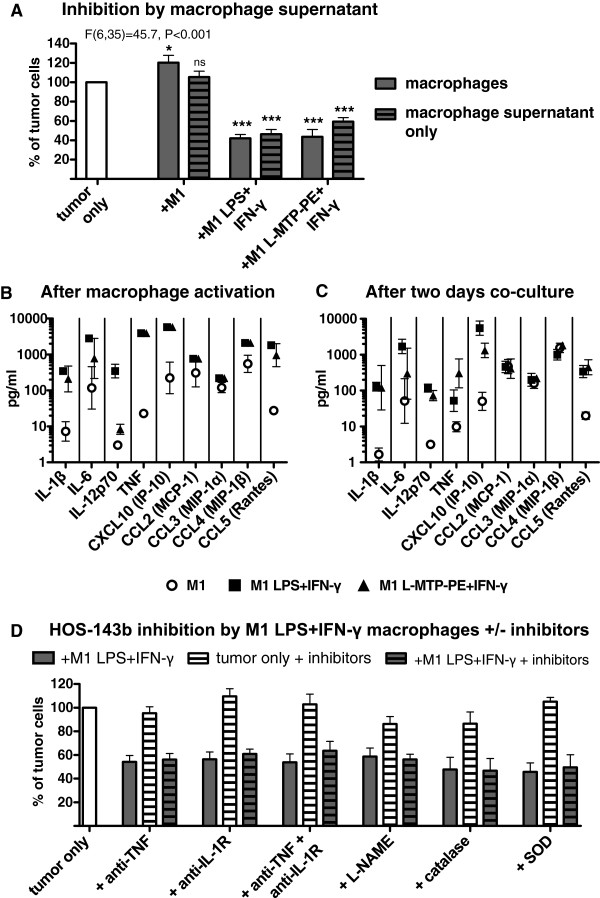
**Tumor growth inhibition by activated M1-like macrophages is mediated by soluble factors. (A)** HOS-143b cells were incubated with M1-like macrophages (pre-activated +/− LPS + IFN-γ or L-MTP-PE + IFN-γ) (no pattern) or with cell culture supernatant from these macrophages (hatched pattern) for two days. Tumor cell numbers were analyzed relative to the control (ANOVA and Dunnett’s post test n = 3–8). The cytokine/chemokine profile of M1-like macrophages was assessed in cell-free supernatants obtained **(B)** after macrophage activation or **(C)** after macrophage activation and subsequent two-day co-culture with HOS-143b cells. Data of IL-1β, IL6, IL-12p70, TNF-α, CXCL10 (IP-10), CCL2 (MCP-1), CCL3 (MIP-1α), CCL4 (MIP-1β) and CCL5 (Rantes) were acquired by Luminex assays (n = 2–3). There was no cytokine/chemokine production by tumor cells alone. Compared to activated macrophages alone the co-culture with tumor cells did not enhance cytokine/chemokine production (data not shown). **(D)** HOS-143b cells were incubated with/without LPS + IFN-γ-activated M1-like macrophages in the presence or absence of inhibitors against TNF-α (by neutralizing antibody and soluble TNF receptor), IL-1 receptor (by IL-1Ra), TNF-α and IL-1 receptor, nitric oxide (by L-NAME) or reactive oxygen species (by catalase and SOD). For each set of inhibitor experiments (n = 3–6) tumor cell numbers of the following conditions are depicted: after co-culture with activated macrophages (filled bar), tumor cells alone with inhibitors (white bar hatched pattern), after co-culture with activated macrophages with inhibitors (filled bar hatched pattern), analyzed relative to tumor cells only without inhibitors (white bar). There was no significant difference between co-cultures with and without inhibitors, or between tumor cells alone and co-cultures with inhibitors, whereas differences between tumor cells alone and co-cultures with/without inhibitors were statistically significant (P < 0.05, ANOVA and Bonferroni’s post test).

Next, the mechanisms involved in the strong anti-tumor effect of LPS + IFN-γ–activated and L-MTP-PE + IFN-γ–activated M1-like macrophages were investigated. Incubation of osteosarcoma cells in medium with cell-free supernatant of activated M1-like macrophages reduced tumor cell growth to similar levels as activated M1-like macrophages themselves (Figure 4, panel A). In contrast, supernatant from non-activated M1-like macrophages did not reduce tumor cell growth. The inhibitory effect of macrophage supernatant was dose-dependent (Additional file 1: Figure S1, panel B). These data indicate that both LPS + IFN-γ–activated and L-MTP-PE + IFN-γ–activated M1-like macrophages produced soluble factors that inhibited osteosarcoma cell growth. Therefore we measured the levels of soluble factors produced by activated macrophages alone and after two-day co-culture with the tumor cells. Activation of M1-like macrophages with LPS + IFN-γ enhanced the production of the pro-inflammatory cytokines IL-1β, IL-6, IL-12p70, TNF-α, CXCL10 (IP-10) and CCL5 (Rantes), while CCL2 (MCP-1), CCL3 (MIP-1α) and CCL4 (MIP-1β) remained unchanged amongst a panel of 27 cytokines and chemokines tested (Figure 4, panel B). L-MTP-PE + IFN-γ–activated M1-like macrophages displayed a similar cytokine profile except for lower levels of IL-12p70.

Since TNF-α was reported to be able to confer anti-tumor effects [29] and was also produced by both LPS + IFN-γ–activated and L-MTP-PE + IFN-γ–activated M1-like macrophages during the co-culture (Figure 4, panel C), a role for TNF-α in the inhibition of osteosarcoma cell growth was examined. Blocking of TNF-α during the co-culture of macrophages and tumor cells by the soluble TNF receptor etanercept combined with the TNF-α neutralizing antibody adalimumab did not prevent the inhibition of cell growth of HOS-143b and OHS cells by LPS + IFN-γ–activated M1-like macrophages or supernatants derived from these macrophages (Figure 4, panel D and data not shown)*.* Blocking of TNF-α did also not prevent the inhibiting effects of L-MTP-PE + IFN-γ–activated M1-like macrophages (data not shown). Moreover, blocking of IL-1 receptor, combined blocking of TNF-α and IL-1 receptor, or inhibition of nitric oxide and reactive oxygen species did not significantly interfere with the inhibition of tumor cell growth by activated macrophages (Figure 4, panel D). None of the tested inhibitors affected tumor cell growth as compared to tumor cells incubated in the absence of inhibitors.

These results indicate that the inhibition of osteosarcoma cell growth by activated M1-like macrophages was mediated by soluble factors induced by macrophage activation in a TNF-α/IL-1–independent manner.

### IL-10–stimulated M2-like macrophages can inhibit growth of osteosarcoma cells in an antibody-dependent manner

**Figure 5:**
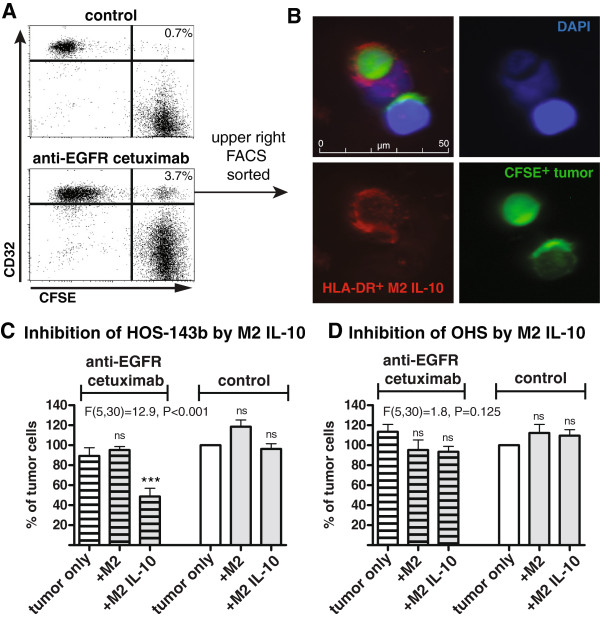
**Antibody-dependent tumor cell growth inhibition by IL-10–polarized M2-like macrophages. (A)** CFSE-labeled HOS-143b cells coated with anti-EGFR cetuximab or non-binding anti-CD20 rituximab were incubated with IL-10–stimulated M2-like macrophages for two hours. Cell conjugate formation was evaluated by flow cytometry, assessing CD32^+^ macrophages acquiring high CFSE fluorescence of the tumor cells. Representative data of two experiments are depicted. **(B)** In one experiment, CD32^+^CFSE^+^ cells (upper right quadrant in lower panel A) were sorted by flow cytometry and examined by Immunofluorescence microscopy, detecting HLA-DR-stained macrophages in red (lower left), CFSE^+^ tumor cells in green (lower right) and DAPI-stained cell nuclei in blue (upper right) and composites (upper left). **(C)** HOS-143b (n = 4–7) and **(D)** sOHS (n = 5–8) cells were coated with cetuximab (hatched pattern) or rituximab (control, no pattern) and incubated with M2-like macrophages pre-stimulated with or without IL-10. After two days tumor cell numbers were analyzed by ANOVA and Dunnett’s post test.

Both M1-like and M2-like macrophages have been detected in osteosarcoma lesions [4]. Hitherto, IL-10–stimulated M2-like macrophages were unable to inhibit osteosarcoma cell growth. In a previous study it has been shown that IL-10-polarized M2-like macrophages internalized antibody-coated B cell lymphoma cells [28]. Since IL-10–stimulated M2-like macrophages exhibited the highest expression of FcγR in our experiments, we investigated whether these macrophages are able to form cell conjugates with and internalize osteosarcoma cells in an antibody-dependent manner as a potential anti-tumor mechanism. After two-hour co-culture of IL-10–stimulated M2-like macrophages and CFSE-labeled HOS-143b cells, CD32^+^ M2-like macrophages acquiring the fluorescent signal of HOS-143b cells were analyzed by flow cytometry, as similarly described before [28,30]. If HOS-143b cells were coated with the anti-EGFR antibody cetuximab, the percentage of CD32^+^CFSE^+^ macrophages was increased, indicative for cell conjugate formation between macrophages and tumor cells (Figure 5, panel A). Furthermore, FACS-sorted CD32^+^CFSE^+^ macrophages (upper right quadrant) were analyzed by immunofluorescence microscopy and contained tumor cells bound to macrophages as well as tumor cells internalized by macrophages (Figure 5, panel B).

Next, it was examined whether the antibody-dependent interaction between tumor cells and macrophages can result in inhibition of tumor cell growth. Indeed, IL-10–stimulated M2-like macrophages substantially inhibited growth of two osteosarcoma cell lines (2/6) such as HOS-143b to as low as 50% if coated with cetuximab (Figure 5 panel C and data not shown). There was not such an inhibiting effect when HOS-143b cells were treated with the isotype-matched, non-tumor-binding anti-CD20 antibody rituximab. In contrast, IL-10–stimulated M2-like macrophages failed to inhibit cell growth of (or form cell conjugates with) cetuximab-coated OHS cells despite high levels of EGFR expression [31], indicating that additional cell characteristics play a role in determining the sensitivity of osteosarcoma cell lines to antibody-dependent anti-tumor activity (Figure 5, panel D and data not shown). In the absence of macrophages, there was no inhibitory effect by cetuximab on osteosarcoma cells (Figure 5, panel C and D) [31]. Inhibition of tumor cell growth by LPS + IFN-γ–stimulated M1-like macrophages was not further increased by cetuximab (data not shown).

Hence, at least for some cell lines, IL-10–stimulated M2-like macrophages have the potential to inhibit osteosarcoma cell growth in an antibody-dependent manner with similar efficacy as antibody-independent inhibition by activated M1-like macrophages.

## Discussion

In this report, we describe for the first time that human macrophages can interfere with the growth of human osteosarcoma cells. Significant induction of anti-tumor activity of human M1-like macrophages by liposomal muramyl tripeptide required co-stimulation with pro-inflammatory IFN-γ. Inhibition of osteosarcoma cell growth by activated M1-like macrophages was mediated by soluble factors which were induced upon macrophage activation before interaction with tumor cells. In addition, we report that IL-10–polarized M2-like macrophages could exert anti-tumor activity against some osteosarcoma cell lines in an antibody-dependent manner.

More than 100 years ago it has been observed by Busch, Fehleisen, Bruns and others that bacterial infections can result in tumor regression accompanied by febrile inflammatory responses which presumably mediated the anti-tumor effects [32,33]. These findings pioneered the first extensive immunotherapy of bone sarcoma patients by Coley, administering heat-inactivated bacterial preparations with considerable but disputed remission rates. The anti-tumor effect was probably, at least in part, linked to the pro-inflammatory response of innate immune cells such as macrophages to bacterial constituents like LPS [33,34]. Another bacterial cell wall component, muramyl dipeptdide (MDP) has originally been discovered as the minimal (synthetically-derived) moiety of peptidoglycan which can substitute for mycobacteria in Freund’s complete adjuvant [35]. MTP-PE is a lipophilic, synthetic derivate of MDP which has low toxicity and enhanced macrophage-activating properties if incorporated in liposomes (L-MTP-PE) [21]. To mimic bacterial infections and trigger macrophage activation, L-MTP-PE has been included in the treatment of osteosarcoma patients [1]. Our observation that the anti-tumor effect of L-MTP-PE–stimulated macrophages was dependent on IFN-γ is noteworthy in this respect. IFN-γ was originally described as macrophage-activating factor [36]. ‘Priming’ of macrophages by IFN-γ may enhance liposome uptake and improve the response to bacterial components by, for instance, intracellular NOD2, which is the receptor for MDP and presumably MTP-PE [21,37-39]. The significance of IFN-γ observed in our experiments reproduces previous studies using different tumor cells which showed that activation of human/murine monocytes/macrophages by L-MTP-PE was enhanced by simultaneous or preceding stimulation with IFN-γ [17,21,38]. Furthermore, addition of IFN-γ to L-MTP-PE was reported to improve survival and inhibit metastases in murine renal adenocarcinoma [40]. Altogether, the clinical efficacy of L-MTP-PE addition in the treatment of osteosarcoma patients may be improved by the inclusion of a macrophage-priming signal like IFN-γ.

This raises the question how such a macrophage-priming factor could be safely introduced in the osteosarcoma microenvironment. IFN-γ levels could be increased by local or systemic IFN-γ therapy as applied in patients with cancers or mycobacterial infections [41,42]. To target the same macrophages with IFN-γ as with L-MTP-PE, IFN-γ could be incorporated in MTP-PE-containing liposomes which are then both efficiently internalized by phagocytic cells such as tumor-resident or tumor-infiltrating macrophages. This approach is supported by murine studies in which the incorporation of IFN-γ into MTP-PE–containing liposomes enhanced the tumoricidal activity of macrophages as compared to liposomal MTP-PE alone [21,43]. Alternatively, lymphocytes such as NK cells activated to secrete IFN-γ and recruited to tumor sites might enhance local IFN-γ production [44].

Inhibition of osteosarcoma cell growth was mediated by soluble factors which were produced by activated M1-like macrophages before interaction with the tumor cells. It is noteworthy that the macrophages themselves as well as their secreted factor reached a maximal effect on inhibiting tumor cell numbers to about 50%. Inhibition of cell growth required time and became only evident after more than one day. Altogether these data suggest that the inhibitory factor may either limit growth of the tumor cells to a certain maximal cell density or that this factor has a delayed cytotoxic effect. However, because a cytotoxic effect is expected to become evident sooner, an anti-proliferative effect of this factor is more likely. Similar to our results, inhibition of colorectal cancer cells by an unidentified soluble factor of macrophages has recently been reported [45].

In our experiments, bacterial and pro-inflammatory stimuli induced the strongest inhibition of tumor cell growth by M1-like macrophages. Therefore, such macrophage-activating therapies may primarily be effective in tumor types that contain M1-like macrophages [14,15]. Most tumors contain high numbers of potentially ‘pro-tumor’ immune regulatory M2-like macrophages. The successful adjuvant therapy with Bacillus Calmette-Guérin in patients with bladder cancer may involve the activation of pro-inflammatory M1-like macrophages, but might be negatively influenced by infiltrating M2-like macrophages [46,47]. Therefore, several studies have considered depleting macrophage numbers or inhibiting macrophage recruitment to the tumor [11,48,49]. Instead, in our experiments, IL-10–polarized M2-like macrophages could be induced to inhibit osteosarcoma cell growth if the tumor cells were coated with the therapeutic anti-EGFR antibody cetuximab. Antibody-dependent cell conjugate formation and inhibition of tumor cell growth were only observed for half of the osteosarcoma cell lines despite significant EGFR expression [31]. Hence, to improve antibody-dependent anti-tumor activity by M2-like macrophages, it would be required to elucidate additional parameters besides surface antigen expression that determine inhibition of tumor cell growth by macrophages. Expression of CD47 on tumors cells has been described to block phagocytic function by binding to SIRP1α expressed on phagocytic cells [50]. However, CD47 gene expression was not significantly different between the cell lines (inhibited or not inhibited by M2-like macrophages), as concluded from previously published genome-wide gene profiling data of osteosarcoma cell lines [4] (data not shown).

The potential of antibody-dependent anti-tumor activity by macrophages has been shown to mediate anti-tumor responses in murine lymphoma models [51,52]. In humans, the addition of rituximab therapy to patients with follicular lymphoma can counteract the non-favorable prognostic factor of high macrophage counts in the tumor [53]. We have previously demonstrated that the cytotoxic activity of NK cells can be enhanced and directed to osteosarcoma cells by anti-EGFR cetuximab [31]. Since macrophages abundantly infiltrate osteosarcoma lesions, antibody-dependent inhibition of osteosarcoma cell growth by macrophages may be an additional anti-tumor mechanism of cetuximab.

The recent finding that anti-CD40 therapy can induce anti-tumor activity in mice and humans independently of T cells but presumably via activating macrophages has revived the role of macrophages in anti-tumor responses [54]. Overall, activation of macrophages by e.g. L-MTP-PE in the presence of IFN-γ, and/or treatment with tumor-reactive antibodies may in particular be advantageous in tumors like osteosarcoma that have a high content of infiltrating macrophages.

## Competing interests

The authors declare that they have no competing interests.

## Authors’ contributions

JP, KK, EV, SS, SM, MK performed experiments and analyzed data. EV and WJ generated and provided liposome preparations. JW verified cell line identities. JP, KK, MT, AC, RE, WJ, AL and MS participated in study conception and data interpretation. MT, RE, AL and MS coordinated this study. JP, AL and MS wrote the manuscript. All authors read and approved the final manuscript.

## Supplementary Material

Additional file 1: Figure S1 Inhibition of tumor cell growth by activated M1-like macrophages is dose-dependent. (A) HOS-143b and OHS cells were incubated with increasing numbers of LPS+IFN-γ–activated M1-like macrophages as indicated by the macrophage:tumor ratios from 0 to 20. (B) HOS-143b and OHS cells were incubated with increasing amounts of cell-free culture supernatant of LPS+IFN-γ and L-MTP-PE+IFN-γ–activated M1-like macrophages as indicated by the percentage of culture supernatant present during tumor cell culture. Of note, supernatant from LPS+IFN-γ-activated M1-like macrophages was slightly more potent in tumor growth inhibition than supernatant of L-MTP-PE+IFN-γ activated M1-like macrophages. Click here for file
