# Supplementary material for: Macrophages inhibit human osteosarcoma cell growth after activation with the bacterial cell wall derivative liposomal muramyl tripeptide in combination with interferon-γ
Source: J Exp Clin Cancer Res. 2014 Mar 10;33(1):27. doi: 10.1186/1756-9966-33-27 (PMC4007518; doi:10.1186/1756-9966-33-27)
Supplement: Additional file 1: Figure S1 — Inhibition of tumor cell growth by activated M1-like macrophages is dose-dependent. (A) HOS-143b and OHS cells were incubated with increasing numbers of LPS+IFN-γ–activated M1-like macrophages as indicated by the macrophage:tumor ratios from 0 to 20. (B) HOS-143b and OHS cells were incubated with increasing amounts of cell-free culture supernatant of LPS+IFN-γ and L-MTP-PE+IFN-γ–activated M1-like macrophages as indicated by the percentage of culture supernatant present during tumor cell culture. Of note, supernatant from LPS+IFN-γ-activated M1-like macrophages was slightly more potent in tumor growth inhibition than supernatant of L-MTP-PE+IFN-γ activated M1-like macrophages. [file 1756-9966-33-27-S1.docx]

**MS:  8974494011141216**
Pahl - Macrophages inhibit human osteosarcoma cell growth after activation with the bacterial cell wall derivative liposomal muramyl tripeptide in combination with interferon-gamma


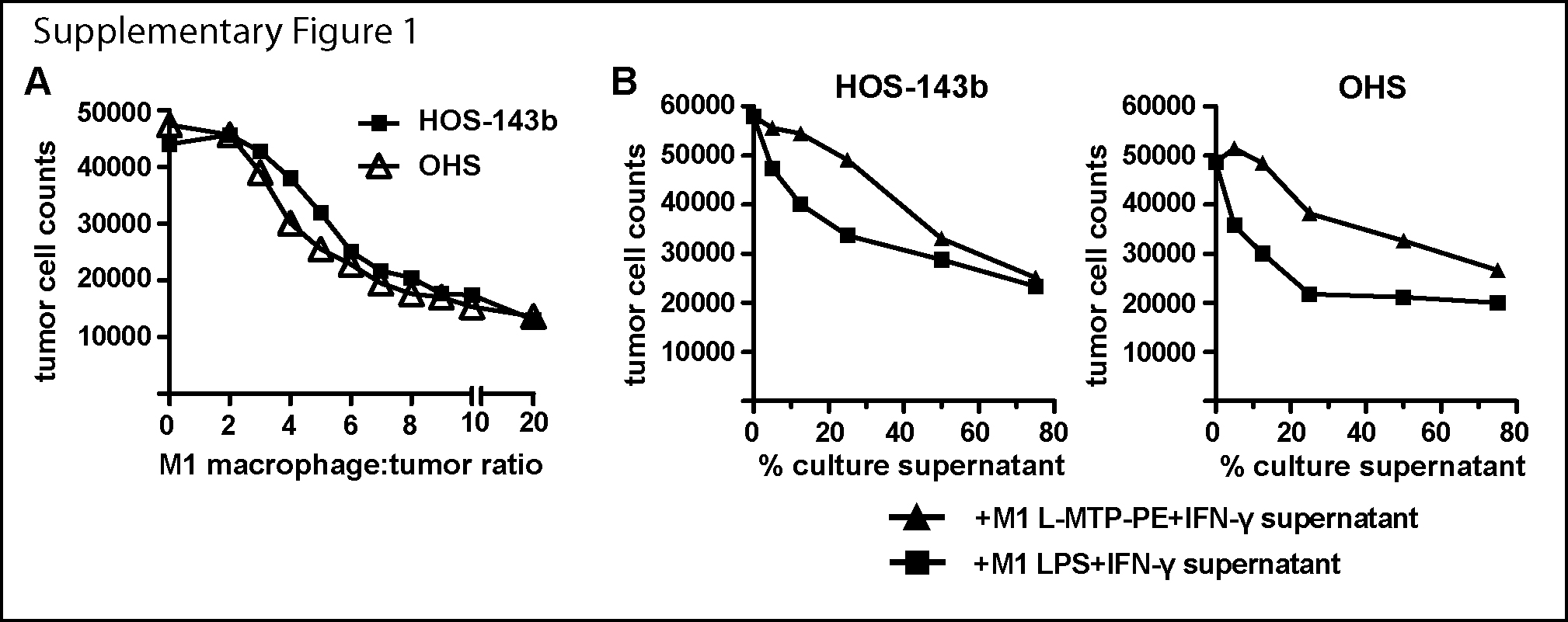


**Inhibition of tumor cell growth by activated M1-like macrophages is dose-dependent**

**(A)** HOS-143b and OHS cells were incubated with increasing numbers of LPS+IFN-γ–activated M1-like macrophages as indicated by the macrophage:tumor ratios from 0 to 20. **(B)** HOS-143b and OHS cells were incubated with increasing amounts of cell-free culture supernatant of LPS+IFN-γ and L-MTP-PE+IFN-γ–activated M1-like macrophages as indicated by the percentage of culture supernatant present during tumor cell culture. Of note, supernatant from LPS+IFN-γ-activated M1-like macrophages was slightly more potent in tumor growth inhibition than supernatant of L-MTP-PE+IFN-γ activated M1-like macrophages.
